# Trace metal ions are strongly associated with the structural variation in vineyard soil microbial communities along altitude gradients

**DOI:** 10.3389/fmicb.2026.1824682

**Published:** 2026-07-08

**Authors:** Yashan Li, Xiaoxiong Bai, Xuan Yu, Bingbing Duan, Wanzhi Li, Liang Zhang, Jiakui Wang, Cili Yangzong, Lijun Nan, Xu Liu

**Affiliations:** 1 College of Enology, Northwest A&F University, Yangling, Shaanxi, China; 2 College of Agriculture, Chuxiong Normal College, Chuxiong, Yunnan, China; 3 College of Forestry, Northwest A&F University, Yangling, Shaanxi, China; 4 College of Enology and Horticulture, Ningxia University, Yinchuan, Ningxia, China; 5 Shangri-La Winery Co., LTD., Shangri-La, Yunnan, China; 6 Dêqên Tibetan Autonomous Prefecture Academy of Agricultural Sciences, Shangri-La, Yunnan, China

**Keywords:** altitude gradients, assembly processes, co-occurrence network, soil microbial communities, vineyards, *α*-diversity

## Abstract

Microorganisms play a crucial role in soil ecosystems by facilitating nutrient cycling and enhancing soil fertility, which in turn significantly affects plant growth. However, current knowledge on changes in soil microbial communities and their drivers in vineyards under anthropogenic management along altitude gradients is still limited. To address this gap, we employed high-throughput sequencing to examine variations in microbial community composition, diversity, co-occurrence networks, and assembly processes in vineyard soils across an altitude gradient (2,017–2,738 m), and further identified the important correlates associated with these variations. The results indicate that altitude gradients significantly shape the composition and diversity of microbial communities in vineyard soils (*p* < 0.05). With increasing altitude gradient, the relative abundance of the bacterial phyla *Bacteroidetes* and *Entotheonellaeota* increases significantly, while that of *Gemmatimonadetes* decreases significantly. The fungal phyla *Ascomycota*, *Olpidiomycota*, and *Chytridiomycota* reach their highest levels at elevated altitudes. Furthermore, the *α*-diversity of microbial communities, bacterial network complexity, and the stochastic processes governing both bacterial and fungal communities all exhibit unimodal patterns. Notably, the complexity of the fungal network progressively increases with altitude gradient, primarily through an increase in the fraction of negative correlations among fungal taxa, which may represent a potential ecological strategy for enhancing community stability or resilience to changing environmental conditions. A pronounced distance-decay pattern is evident in microbial communities. Total nitrogen (TN) and exchangeable magnesium (EMg) are identified as important correlates influencing community composition. Hierarchical partitioning reveals that trace metal ions account for a relatively larger fraction of community variability than soil nutrients in our analysis. Available potassium (AK), available phosphorus (AP), and total phosphorus (TP) are associated with the complexity of both bacterial and fungal networks. Additionally, available zinc (AZn) is identified as an important correlate of fungal network complexity. Stochastic processes governing microbial communities are primarily driven by drift and dispersal limitation, with available copper (ACu) and available iron (AFe) showing stronger explanatory power for the assembly of bacterial and fungal communities. In summary, interactions between fungal taxa play a crucial role in enhancing their environmental adaptability, and trace metal ions are among the most important correlates of bacterial and fungal community structure along altitude gradients in vineyards. These findings provide a theoretical basis for future efforts aimed at understanding and potentially managing soil microbial communities in vineyard systems, although a direct link to soil quality improvement requires further investigation.

## 1 Introduction

Vineyards, as integral components of agricultural ecosystems, not only serve as sites for cultivating high-value crops but also as habitats for diverse soil microbial communities (Bettenfeld, 2022; Coller et al., 2019). Soil microbes significantly enhance vineyard functioning by improving soil fertility through organic matter decomposition and nutrient cycling, thereby promoting grape quality (Köberl et al., 2020; Li et al., 2024). Evidence indicates that microbial diversity can enhance soil quality (Liu et al., 2022; Liu W. et al., 2024). Furthermore, microbial communities are highly structured, with interactions among different taxonomic groups giving rise to complex networks via matter and energy exchange (Wagg et al., 2019; Wang X. et al., 2023). The formation of such microbial networks facilitates complementarity in soil ecological functions, further improving soil quality (Wang X. et al., 2023; Kang et al., 2023). Current research suggests that both deterministic and stochastic processes play crucial roles in governing microbial communities assembly, thereby coupling microbial diversity with ecosystem functionality (Wang J. et al., 2023; Zhu et al., 2024).

Climate is a critical factor influencing wine style, regional terroir, and quality (Liu et al., 2019). Global climate change, characterized by warming and drought, directly affects the soil micro-ecological environment in vineyards (Zeng et al., 2023). These impacts subsequently affect growing practices, management strategies, and ultimately wine quality. Altitude gradients encompass variations in multiple climatic factors, including temperature, humidity, and light, which change at a rate 1,000 times faster than that observed across latitudinal gradients (Li et al., 2018). Therefore, investigating variations in vineyard soil microbial communities in vineyard soils and their associated environmental drivers across altitude gradients is of considerable importance for addressing the challenges posed by climate change.

Extensive research has demonstrated that altitude gradients significantly influence the physicochemical properties of soil, which in turn are associated with the composition, structure, and functionality of soil microbial communities (Zhang and Xu, 2023; He et al., 2024a; Ji et al., 2022). Across altitude gradients, microbial diversity exhibits several patterns, including monotonic decreases, stair-step trends, and unimodal distributions (He et al., 2024a; Lanzén et al., 2016; Li et al., 2018; Ren et al., 2018; Yasir et al., 2015; Zhao et al., 2020). For instance, Ren et al. (2018) found that altitude gradient significantly impacts bacterial diversity in Mount Taibai, while its influence on fungal diversity is relatively minor. The primary factors associated with microbial diversity in this context include the plant Shannon index, soil organic carbon, and total nitrogen. Li et al. (2018) discovered that the *α*-diversity of soil bacteria in Mount Gongga follows a stair-step decline pattern with increasing altitude gradient, with soil pH identified as an important correlate of bacterial diversity. Similarly, Chen et al. (2022) reported that both bacterial and fungal community diversity and network complexity decline significantly at higher altitudes on the Tibetan Plateau. Li et al. (2020) suggested that bacterial networks tend to be more complex at lower altitudes, while fungal networks exhibit the opposite trend. Environmental factors, such as soil pH, and biological factors, including community composition, are strongly correlated with bacterial network formation, while biodiversity plays an important role in shaping fungal networks.

Further studies have demonstrated that stochastic processes predominantly govern the assembly of arbuscular mycorrhizal (AM) fungal communities along altitude gradients, with their relative importance increasing with altitude (Liu et al., 2023). Li et al. (2020) highlighted that stochastic processes, such as dispersal limitation and drift, largely control the spatiotemporal dynamics of soil bacterial communities in northern mountain ecosystems. In contrast, other studies have shown that deterministic processes dominate soil bacterial assembly at altitudes of 1,800–4,100 m (Li et al., 2018). Notably, research on soil properties and microorganisms across altitude gradients has primarily focused on natural landscapes, including grasslands (Chen et al., 2022), areas with various vegetation types (Kang et al., 2023; Li et al., 2020, 2018), and forests (Ji et al., 2024; Liu et al., 2023). These studies indicate that microbial community characteristics vary unpredictably with altitude, potentially due to differences in climate, altitude range, and soil properties. However, vineyard soils subject to intensive anthropogenic management differ significantly from natural landscapes. Thus, it remains uncertain whether the patterns of microbial community diversity, co-occurrence network features, and assembly processes observed along altitude gradients in natural ecosystems can be directly extended to vineyard soils.

Soil factors, such as pH and organic matter content, are widely recognized as key factors associated with soil microbial community structure (Philippot et al., 2024; Zheng et al., 2019). These factors not only regulate microbial growth and metabolism but also shape microbial interactions and influence the assembly processes and stability of microbial communities (Ratzke et al., 2020; Zheng et al., 2019). Trace metal ions are increasingly recognized as critical correlates of biogeochemical processes (Moreno-Jiménez et al., 2023; Radujković et al., 2021). Dai et al. (2023) underscored the significance of trace metal ions in shaping soil microbial diversity and community composition, noting that their effects on bacterial communities are stronger than on fungal communities. Moreover, the correlation between the available trace metal ions and the microbial community is more pronounced than that of the total trace metal ions. Studies in maize soils have found that trace metal ions, particularly iron and copper, exhibit stronger explanatory power for microbial community shifts than macronutrients (Peng et al., 2022). However, the relative contribution of soil pH, nutrients, and trace metal ions to microbial variability in vineyard soils across altitude gradients remains unclear.

Given these considerations, it is essential to recognize that findings from natural ecosystems may not be directly applicable to anthropogenically managed vineyards. Unlike natural mountain systems characterized by diverse plant communities and minimal human interference, vineyards are intensive agricultural systems dominated by a single crop (*Vitis vinifera* L.) and subject to regular management practices such as tillage, fertilization, irrigation, and pesticide application. These anthropogenic activities can profoundly alter soil physicochemical properties, potentially masking or modulating natural altitude gradient effects observed in pristine environments. Moreover, while soil pH and nutrients are well-established correlates in natural systems, the role of trace metal ions-which can accumulate or be redistributed due to agricultural practices (e.g., copper-based fungicides)-remains critically understudied in vineyard contexts. Consequently, a direct extrapolation from natural altitude gradients to vineyard systems is not appropriate, underscoring the pressing need for system-specific investigations.

The Shangri-La wine region in Yunnan Province exhibits substantial altitude variation, with Cabernet Sauvignon (*Vitis vinifera* L.) cultivated mainly across a range from the dry and hot river valleys at 1,900 m to the cool and secluded valleys at 2,800 m. This unique distribution of vineyards along the altitude gradients provides an exceptional natural setting for investigating the dynamics of soil microbial communities.

In this study, we aim to systematically explore the composition, diversity, network structure, and assembly processes of soil microbial communities across different altitude gradients in the Shangri-La wine region, with a particular focus on identifying the key correlations associated with these community changes. Our specific objectives were: (1) to characterize the variations in soil microbial community diversity, network characteristics, and assembly processes along the altitude gradients, and (2) to identify the environmental factors associated with changes in soil microbial community structure.

We hypothesized that: (1) microbial diversity, network complexity, and assembly processes would exhibit unimodal patterns along the altitude gradient; and (2) given their essential roles in microbial metabolism and stress regulation, trace metal ions would show stronger associations with microbial community composition and assembly mechanisms than basic soil nutrients.

The findings of this study will not only enhance our understanding of the soil microbial ecology of soils along altitude gradients, but also provide a scientific basis for soil management and sustainability in vineyards.

## 2 Materials and methods

### 2.1 Study sites

**Figure 1:**
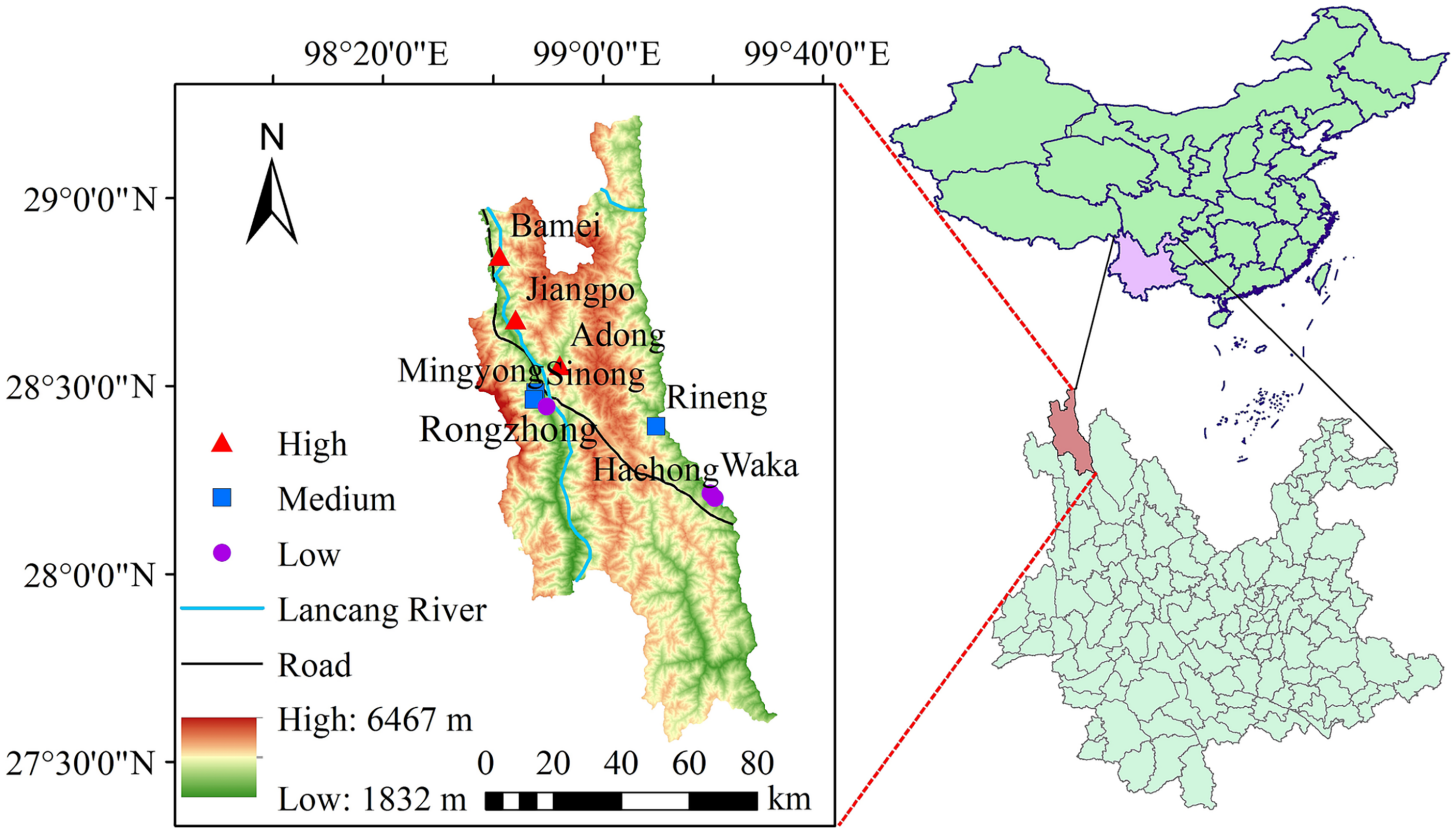
Locations of the sampled vineyards.

The study area is located in Deqin County, Diqing Prefecture, Yunnan Province, Southwestern China. Vineyards at different altitudes were categorized into three altitude gradients: low (1,900–2,200 m), medium (2,200–2,500 m), and high (2,500–2,800 m). Three representative vineyards were randomly selected from each altitude gradient (Figure 1). Detailed information on the vineyards is provided in Table 1.

**Table 1:** Sampling vineyards geographic information.

Vineyard	Altitude m	Latitude	Longitude	Altitude gradient level
Jiangpo	2,738	28°40′57”	98°44′12”	High
Bamei	2,725	28°51′06”	98°41′15”	High
Adong	2,608	28°33′41”	98°52′13”	High
Sinong	2,362	28°29′25”	98°47′51”	Medium
Rineng	2,307	28°23′59”	99°09′33”	Medium
Mingyong	2,272	28°28′16”	98°47′31”	Medium
Rongzhong	2,117	28°27′06”	98°49′52”	Low
Hachong	2058	28°12′29”	99°20′07”	Low
Waka	2017	28°13′12”	99°19′22”	Low

### 2.2 Soil sampling and determination of soil physicochemical indicators

Sampling was conducted in mid-August 2021. In each vineyard, six sampling plots (10 m × 10 m) were randomly selected, with each plot serving as one biological replicate, resulting in six replicates per vineyard and a total of 54 samples across the nine vineyards. Sampling was performed in the inter-row space, 30–40 cm away from the vine. In each plot, soil samples were collected from five points following an S-shaped pattern. During sampling, stones, litter, and other debris from the soil surface were removed and topsoil was collected from a depth range of 0–20 cm using a soil auger. The five subsamples from each plot were thoroughly mixed, quickly passed through a 2 mm sieve, and an aliquot was obtained using the quartering method and transferred into pre-prepared sterile and nuclease-free centrifuge tubes (2 mL). In total, six soil samples were taken from each vineyard, for a total of 54 samples from the nine vineyards. These samples were labeled, immediately transported to the laboratory, and stored at −80 °C in an ultra-low temperature freezer for subsequent determination of bacterial and fungal abundance and diversity. At the same time, the remaining soil was air-dried for the analysis of soil physicochemical properties.

Total nitrogen (TN) content was determined using the Kjeldahl method (Bremner and Mulvaney, 1982). Total phoshphorus (TP) and available phosphorus contents were determined following the method of Zhang et al. (2021). Total potassium (TK) and available potassium (AK) were quantified according to Bao (2000). Soil organic matter (SOM) content was measured by the potassium dichromate volumetric method with external heating (Zhang et al., 2020). Nitrate nitrogen (NN) and ammonium nitrogen (AN) were extracted with 2 M KCl and determined using a continuous flow injection analyzer (AA3, SEAL Analytical GmbH, Norderstedt, Germany) (Zhang et al., 2016). Exchangeable calcium (ECa) and exchangeable magnesium (EMg) were extracted by displacement with 1 M ammonium acetate (pH 7) and measured by atomic absorption spectroscopy (PinAAcle 900F, PerkinElmer, USA) (Bao, 2000). The contents of available copper (ACu), available iron (AFe), available zinc (AZn), and available manganese (AMn) in the soil were determined using the DTPA-TEA extraction and flame/graphite furnace atomic absorption spectrometry (900 T AAS, PerkinElmer Inc., Waltham, MA, USA)(Lindsay and Norvell, 1978). The soil electrical conductivity (EC) was measured using a conductivity meter (DDS-11, China). Soil pH was measured using a pH meter (pHS-3C) in a 1.00:2.50 (w/v) soil: water suspension (Zhang et al., 2021).

### 2.3 Soil DNA extraction, PCR amplification and Illumina sequencing

Total soil DNA was extracted from 0.25 g of fresh soil using the OMEGA Soil DNA Kit (Omega Bio-Tek, Norcross, GA, USA), following the manufacturer’s instructions, and stored at −20 °C prior to further analysis. The quantity and quality of extracted DNAs were determined using a NanoDrop NC2000 spectrophotometer (Thermo Fisher Scientific, Waltham, MA, USA) and agarose gel electrophoresis, respectively. For bacterial community analysis, primers targeting the V3-V4 region of the bacterial 16S rRNA gene were used: 338F (5’-ACTCCTACGGGAGGCAGCA-3′) and 806R (5’-GGACTACHVGGGTWTCTAAT-3′). For fungal community analysis, the internal transcribed spacer (ITS) rRNA gene was amplified using primers ITS5 (5’-GGAAGTAAAAGTCGTAACAAGG-3′) and ITS2(GCTGCGTTCTTCATCGATGC). Each sample was labeled with a unique barcode for multiplex sequencing. The PCR reaction mixture contained 5 μL reaction buffer (5×), 5 μL high-GC buffer (5×), 2 μL dNTPs (10 mM), 0.25 μL Q5 high-fidelity DNA polymerase (5 U·μL^−1^) (New England Biolab, UK), 2 μL DNA template, 1 μL (10 μM) of each primer, and 8.75 μL ddH_2_O. The thermal cycling conditions for bacteria were as follows: initial denaturation at 98 °C for 5 min; 25 cycles of 98 °C for 30 s and 53 °C for 30 s, and 72 °C for 45 s; followed by a final elongation step at 72 °C for 5 min. The conditions for fungi were: initial denaturation at 98 °C for 5 min; 28 cycles 98 °C for 30 s and 55 °C for 45 s, and 72 °C for 45 s; followed by a final elongation step of 72 °C for 5 min. The purified PCR products were sequenced on an Illumina MiSeq platform using paired-end 300 bp reads (Illumina Corporation, San Diego, USA).

Raw sequencing data were processed using QIIME2 (version 2021.4). After quality filtering, denoising, and chimera removal using the DADA2 pipeline, amplicon sequence variants (ASVs) were generated. To correct for uneven sequencing depth across samples, the sequencing depth was normalized using cumulative sum scaling (CSS) as implemented in the ‘metagenomeSeq’ R package (Paulson et al., 2013).

### 2.4 Data analysis

Raw ASV tables were filtered before downstream analysis. To minimize the influence of rare or potentially spurious ASVs, those that appeared in fewer than nine samples (i.e., fewer than one-sixth of all samples, *n* = 54) across the entire dataset were removed. This filtering criterion was applied consistently before all subsequent analyses, including diversity calculations, NMDS ordination, distance-decay analysis, distance-based redundancy analysis (dbRDA), co-occurrence network construction, and null-model assembly analysis.

The normalized ASV tables are used for all subsequent analyses. Microbial data were analyzed using R software (version 4.2.1). The ‘vegan’ package was used to calculate microbial diversity indices and to perform non-metric multidimensional scaling (NMDS) based on Bray-Curtis distances, thereby assessing differences in microbial community composition. The ‘geosphere’ and ‘vegan’ packages were used to analyze the distance-decay relations in microbial communities, examining how microbial dissimilarity varies with altitude and geographical distance. dbRDA and hierarchical partitioning (HP) were conducted using the ‘vegan’ and ‘rdacca.hp’ packages to identify the primary soil factors associated with changes in microbial community composition. HP was also employed to explore the relative contributions of soil nutrients and trace metal ions to the shift in microbial community composition.

Co-occurrence network analysis was used to examine the structure and interactions of microbial communities at different altitude gradients. Based on the filtered ASV table, Spearman rank correlations among ASVs were computed, and only robust correlations (|r| > 0.6) with a Benjamini-Hochberg (BH) adjusted *p*-value < 0.01 were used to construct the networks (Benjamini et al., 2006). The choice of |r| > 0.6 as the correlation threshold follows common practice in microbial network analysis (e.g., Barberán et al., 2012; de Vries et al., 2018), as it retains biologically meaningful associations while excluding weak, potentially spurious correlations. The BH-adjusted *p*-value threshold of 0.01 further controls for false positives due to multiple testing. However, we acknowledge that network topology-including metrics such as connectivity, mean degree, and clustering coefficient-can be sensitive to the choice of correlation threshold. While the absolute values of network metrics may vary with different thresholds, the relative patterns across altitude gradients (e.g., unimodal or monotonic trends) remained consistent when we tested alternative thresholds (e.g., |r| > 0.5 or |r| > 0.7; data not shown). The network topology properties, including connectivity, average degree, average path length, diameter, and average clustering coefficient, are quantified using the ‘ggClusterNet’ package. Networks were visualized using Gephi^1^, and subnetworks were extracted for each sample using the ‘subgraph’ function from the ‘igraph’ package. Subnetwork topological parameters were quantified using the ‘network.4’ function from the ‘igraph’ and ‘ggClusterNet’ packages. Multiple regression on matrices (MRM) was performed using the ‘ecodist’ package to determine the contributions of soil factors, including nutrients and soluble trace metal ions, to microbial co-occurrence networks and community assembly processes.

To evaluate microbial community assembly across altitude gradients, null model analysis was applied following the methods of Stegen et al. (2015, 2013). The Beta Nearest Taxon Index (βNTI) and the Raup-Crick Index (RCBray) were calculated to assess community assembly. Ecological processes are classified as either deterministic or stochastic, and were further classified into five categories: homogeneous selection, heterogeneous selection, homogeneous dispersal, dispersal limitation, and drift. Specifically, |βNTI| > 2 indicated deterministic processes (βNTI < −2 for homogeneous selection; βNTI > 2 for heterogeneous selection). |βNTI| < 2 combined with |RCBray| < 0.95 indicated drift, while |βNTI| < 2 and |RCBray| > 0.95 indicated probabilistic dispersal (RCBray < −0.95 for homogenizing dispersal and RCBray > 0.95 for dispersal limitation). The results are visualized using the ‘ggplot2’ package.

A one-way ANOVA followed by a Tukey post-hoc multiple comparison was performed in SPSS version 25 to analyze the effect of altitude gradients on phylum-level relative abundances and diversity indicators. Spearman rank correlation coefficients were calculated to assess the relationship between soil factors and phylum-level abundances, diversity indicators, and network topological properties.

To account for the hierarchical structure of the sampling design-where six plots were nested within each vineyard and three vineyards were nested within each altitude gradient-all statistical analyses were conducted at the plot level (*n* = 54). For analyses testing altitude gradient effects (e.g., one-way ANOVA, dbRDA, null-model analysis), altitude gradient (low, medium, high) was treated as a fixed factor. To avoid pseudoreplication and to control for potential clustering effects due to shared vineyard characteristics (e.g., soil history and local management practices), the following precautions were taken: (1) For one-way ANOVA, residual diagnostics were examined to ensure that no strong vineyard-level clustering effects were present; (2) For dbRDA and MRM, which are permutation-based methods that do not readily accommodate random effects, significance was assessed using 999 permutations.

For all statistical tests, we report both *p*-values and effect sizes. The correlation coefficient *ρ* is reported as the effect size for Spearman correlations, and *R*^2^or independent contribution values are reported for dbRDA, MRM, and hierarchical partitioning.

## 3 Results and analysis

### 3.1 Composition and diversity of bacterial and fungal communities

#### 3.1.1 Comparison at the phylum level

**Figure 2:**
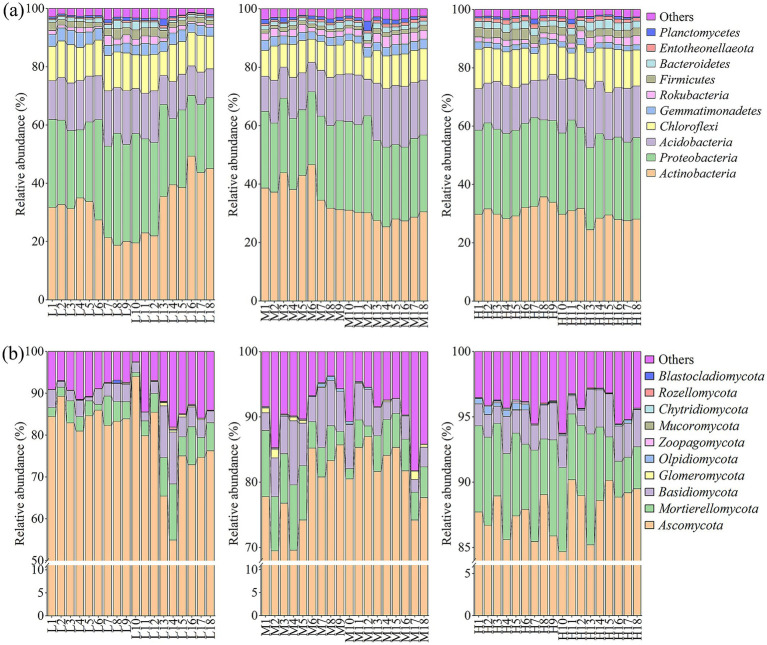
Relative abundances of main soil bacterial **(a)** and fungal **(b)** communities at phylum level in different altitude gradient. L, low; M, medium; H, high.

A total of 241,868 bacterial ASVs and 14,777 fungal ASVs were identified in vineyard soils across different altitude gradients. Bacterial communities were primarily composed of 45 phyla, with the dominant phyla being *Actinobacteria* (31.75%), *Proteobacteria* (28.22%), *Acidobacteria* (15.41%), *Chloroflexi* (11.41%), *Gemmatimonadetes* (2.82%), *Rokubacteria* (2.12%), *Firmicutes* (2.05%), and *Bacteroidetes* (1.75%), each with an average relative abundance exceeding 1% (Figure 2a). The significance of the differences in the relative abundance of soil bacteria at different altitude gradients is shown in Supplementary Table S1. The relative abundance of *Entotheonellaeota* increases significantly with altitude gradient, while the relative abundance of *Gemmatimonadetes* decreases. The relative abundances of *Chloroflexi* and *Firmicutes* were lowest at medium altitude gradient and significantly lower than at low and high altitude gradients, while *Planctomycetes* and *Rokubacteria* were most abundant at the medium altitude gradient.

Fungal communities were dominated by 16 phyla, with *Ascomycota* (82.54%), *Mortierellomycota* (5.30%), and *Basidiomycota* (3.90%) exhibiting the highest relative abundances (Figure 2b). *Ascomycota* was most abundant at the high altitude gradient, where its relative abundance was significantly higher than at the low and medium altitude gradients. Meanwhile, *Basidiomycota* and *Zoopagomycota* peaked at the medium altitude gradient, with *Basidiomycota* being significantly more abundant than at the high altitude gradient and *Zoopagomycota* significantly more abundant than at the lower altitude gradients.

#### 3.1.2 Comparison at the genus level

**Figure 3:**
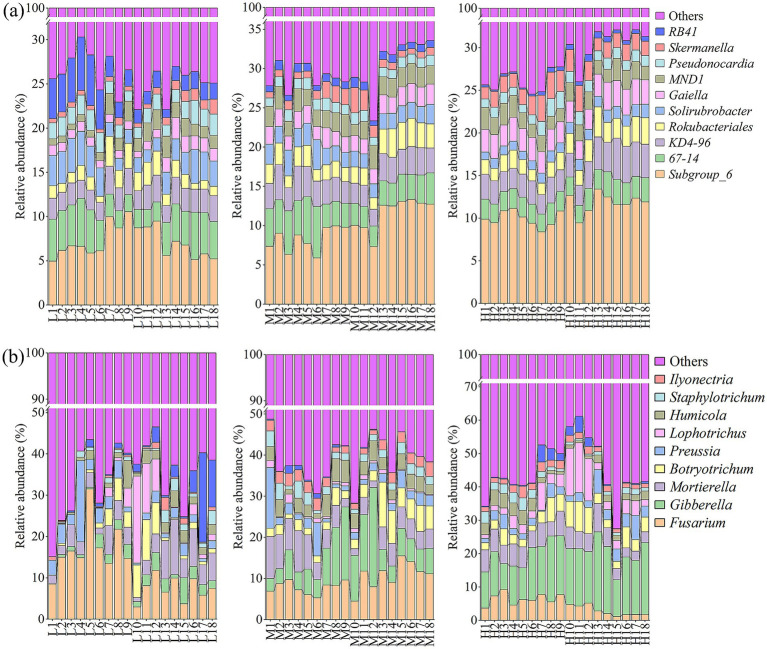
Relative abundances of main soil bacterial **(a)** and fungal **(b)** communities at genus level in different altitude gradients. L, low; M, medium; H, high.

Bacterial communities were primarily composed of 4,216 genera, with the dominant genera being *Subgroup_6* (9.32%), *67–14* (3.31%), *KD4-96* (2.96%), *Rokubacteriales* (2.12%), *Solirubrobacter* (2.05%), and *Gaiella* (2.03%), each with an average relative abundance exceeding 2% (Figure 3a). The significance of the differences in the relative abundances of soil bacterial genera across different altitude gradients is shown in Supplementary Table S2. The relative abundance of *Gaiella* and *Skermanella* significantly increased with altitude gradient, while the relative abundance of *Solirubrobacter* showed a non-significant decrease. The relative abundances of *Subgroup_6* and *KD4-96* were lowest at the low altitude gradient, significantly lower than at the medium and high altitude gradients. In contrast, *Pseudonocardia* and *RB41* were significantly more abundant at the low altitude gradient than that at the medium and high altitude gradients. The relative abundance of *MND1* was highest at the medium altitude gradient, significantly higher than at the other altitude gradients. Similarly, the relative abundance of *Skermanella* was highest at the high altitude gradient, significantly higher than at the other altitude gradients. However, the relative abundances of *67–14* and *Solirubrobacter* were lowest at the high altitude gradient, significantly lower than at the other altitude gradients.

Fungal communities were dominated by 84 genera, with *Fusarium* (8.73%), *Gibberella* (8.73%), and *Mortierella* (5.29%) exhibiting the highest relative abundances (Figure 3b). *Gibberella* was most abundant at the high altitude gradient, significantly exceeding its abundance at the low and medium altitude gradients. In contrast, the relative abundance of *Fusarium* was lowest at the high altitude gradient, significantly lower than at the low and medium altitude gradients. Meanwhile, *Humicola* and *Ilyonectria* peaked at the medium altitude gradient, where their relative abundances were significantly higher than at the other altitude gradients.

#### 3.1.3 Analysis of soil microbial community alpha diversity in different altitude gradients

Altitude gradient significantly affected microbial diversity, with all diversity indices except the Shannon index of bacteria exhibiting a unimodal distribution (Table 2). The Chao1 index, representing bacterial species richness, was highest at the medium altitude gradient and significantly higher than at the high altitude gradient. However, the Shannon index, which measures bacterial diversity, was highest at the low altitude gradient and significantly higher than at the medium and high altitude gradients. For fungi, both the Chao1 richness and Shannon diversity indices peaked at the medium altitude gradient, which were 43.67 and 8.71% higher, respectively, than at the low altitude gradient. Bacterial abundances and diversity were generally higher than those of fungal communities. With the exception of the fungal Shannon index, the range of the other indices were larger at the medium and low altitude gradients but smaller at the high altitude gradient, a pattern consistent with the observed coefficient of variation.

**Table 2:** Alpha diversity of soil bacteria and fungi in different altitude gradients.

Alpha diversity index		Bacteria		Fungi	
		Chao1	Shannon	Chao1	Shannon
Low	Average	6872.70ab	11.44a	467.87b	6.20b
	Maximum value	8290.24	11.76	744.20	7.34
	Minimum value	4999.26	11.14	157.00	4.27
	Range	3290.98	0.62	587.2	3.07
	Standard deviation	946.08	0.17	196.35	0.79
	Coefficient of variation (%)	13.77	1.49	41.97	12.74
Medium	Average	7469.69a	11.30b	672.19a	6.74a
	Maximum value	8801.32	11.64	822.89	7.49
	Minimum value	5334.29	11.01	527.32	6.13
	Range	3467.03	0.64	295.57	1.36
	Standard deviation	1100.47	0.16	82.42	0.41
	Coefficient of variation	14.73	1.42	12.26	6.08
High	Average	6572.15b	11.07c	613.55a	6.48ab
	Maximum value	7861.34	11.24	747.01	6.95
	Minimum value	5333.41	10.90	542.14	5.45
	Range	2527.93	0.34	204.87	1.50
	Standard deviation	728.83	0.09	61.11	0.39
	Coefficient of variation	11.09	0.81	9.96	6.02

Different lowercase letters in the same column indicate significant differences (*p* < 0.05).

#### 3.1.4 NMDS analysis of bacterial and fungal

**Figure 4:**
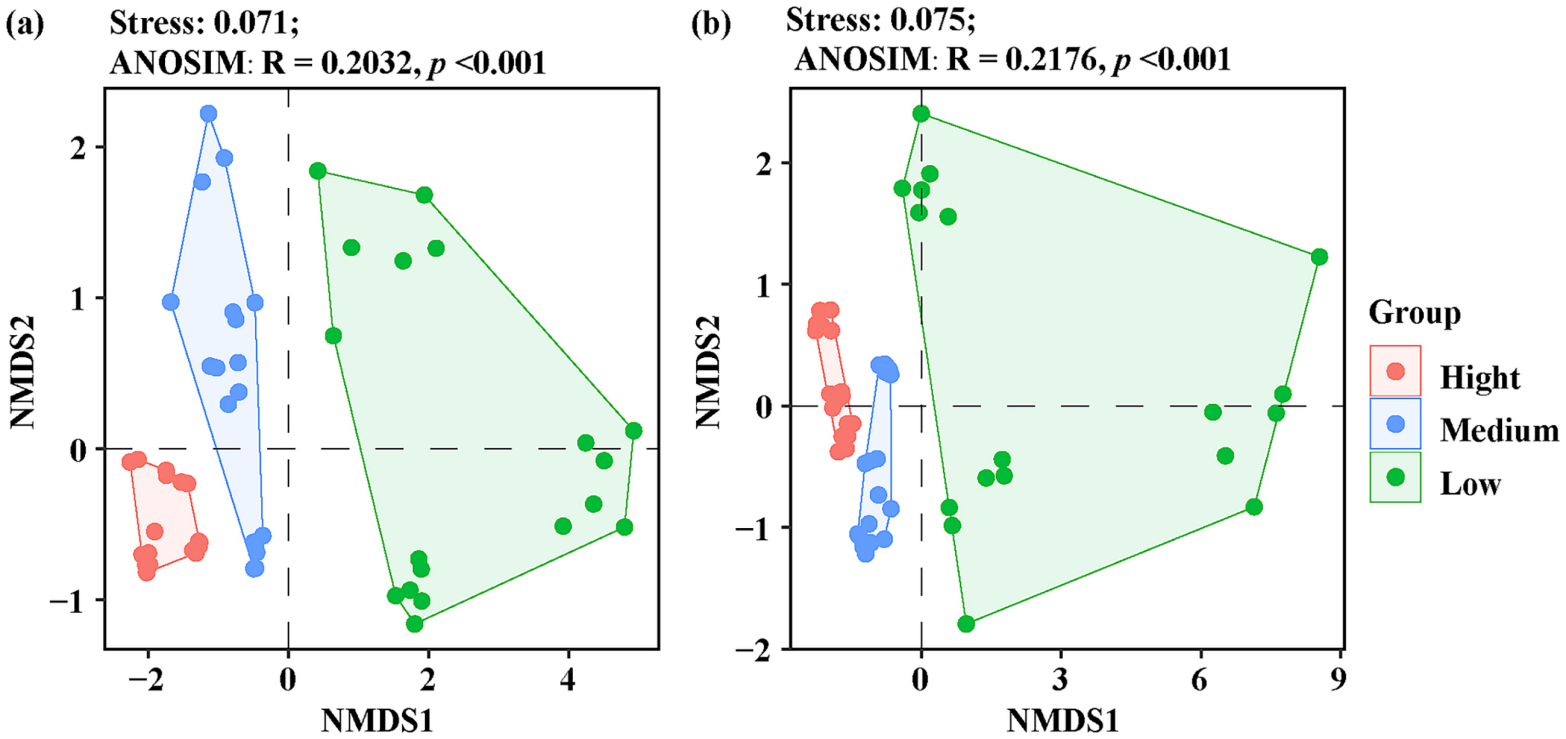
Non-metric multidimensional scaling (NMDS) analysis of soil bacterial **(a)** and fungal **(b)** communities based on Bray-Curtis distances.

Non-metric multidimensional scaling (NMDS) analysis revealed clear clustering of bacterial (Figure 4a) and fungal (Figure 4b) communities along the altitude gradient. Stress values below 0.1 confirmed significant differences in the composition of both bacterial and fungal communities across altitude gradients (*p* < 0.001). Fungal communities exhibited stronger clustering than bacterial communities across altitude gradients.

**Figure 5:**
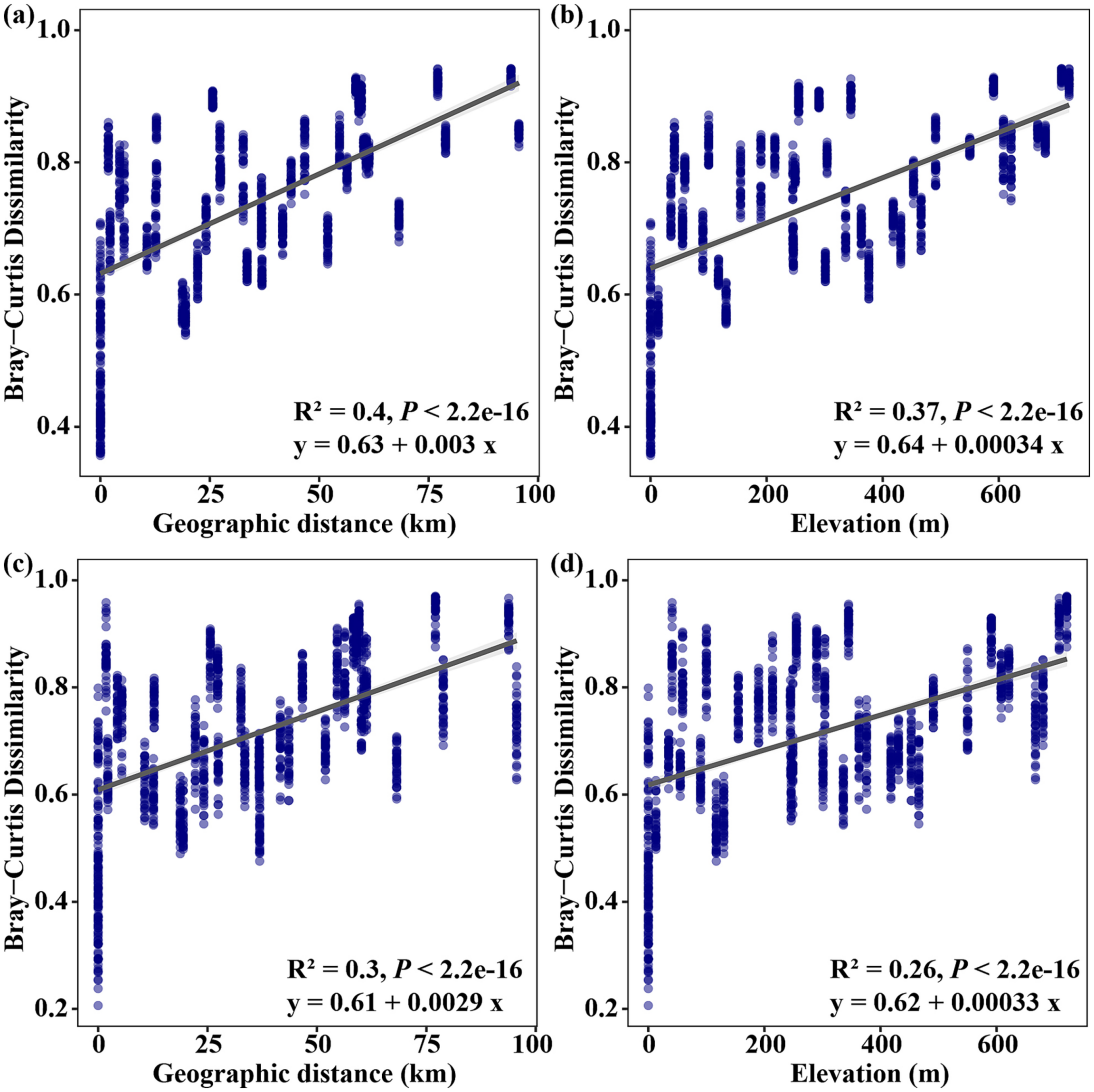
Decay patterns of community Bray–Curtis dissimilarity based on bacterial **(a,c)** and fungal **(b,d)** communities in terms of spatial distance and altitude.

Linear regression between community dissimilarity and geographic distance or altitude revealed a significant distance-decay pattern for both bacterial (Figures 5a,b) and fungal communities (Figures 5c,d) (*p* < 0.001). As geographic distance and altitude increased, community dissimilarity also increased, with more spatially distant samples exhibiting greater differences in community structure. Bacterial communities showed a steeper distance-decay slope compared to fungal communities, indicating that bacterial communities exhibited greater compositional differences with increasing distance.

### 3.2 Relationships between community structure and soil factors

**Figure 6:**
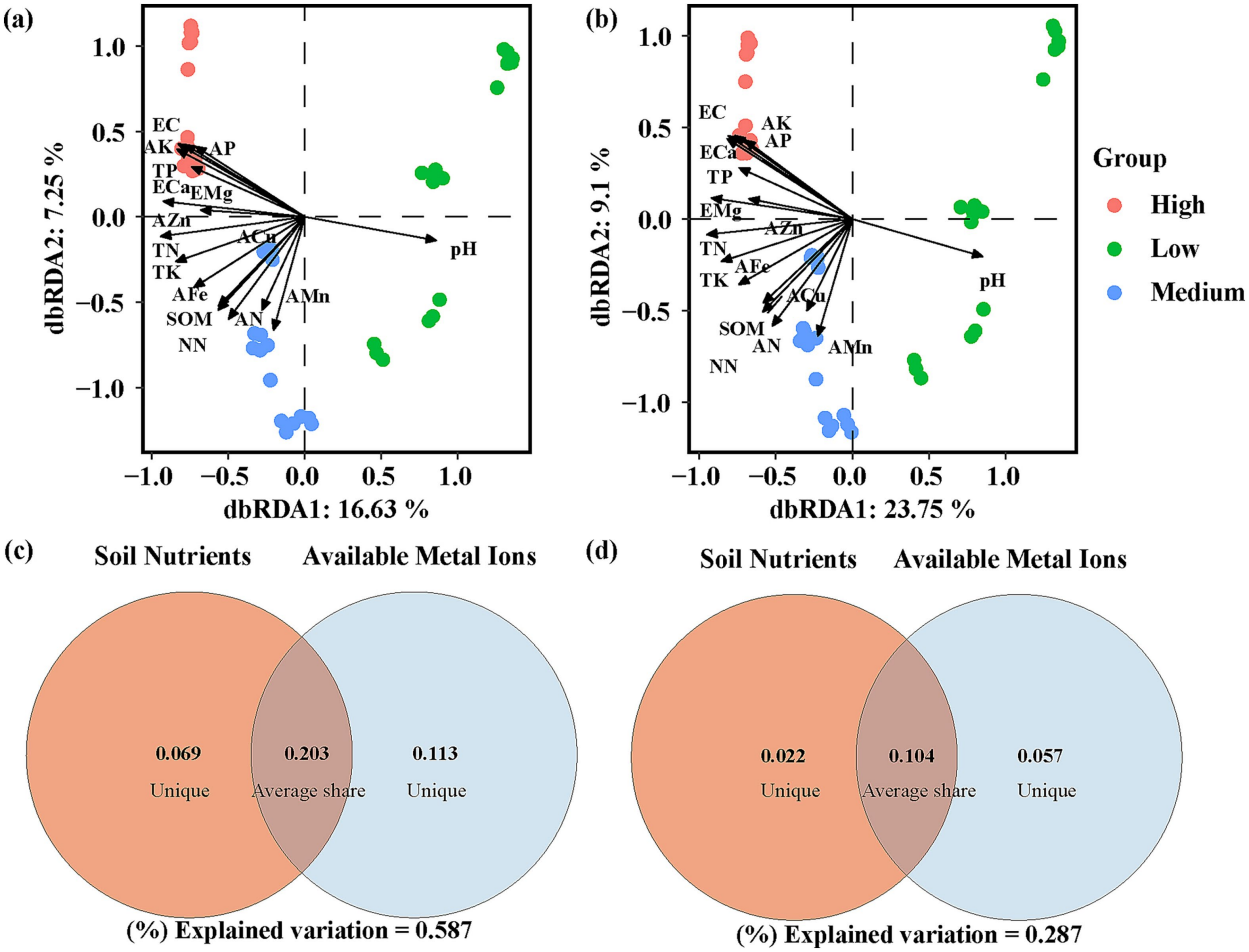
Distance-based redundancy analysis (dbRDA) based on soil bacterial **(a)** and fungal **(b)** community and soil factors. The hierarchical partitioning (HP) of bacterial **(c)** and fungal **(d)** community. EC, Electric conductivity; SOM, Soil organic matter; TN, Total nitrogen; TP, Total phosphorus; TK, Total potassium; NN, Nitrate nitrogen; AN, Ammonium nitrogen; AP, available phosphorus; AK, available potassium; EGa, Exchangeable calcium; EMg, Exchangeable magnesium; AFe, available iron; AMn, available manganese; ACu, available copper; AZn, available zinc.

Redundancy analysis (RDA) showed that the first two axes explained 23.88% of the variation in bacterial community composition and 32.85% of the variation in fungal community composition, with soil factors accounting for a greater proportion of the variation in fungal communities (Figures 6a,b). With the exception of AN, all soil factors significantly influenced microbial community structure (*p* < 0.05) (Table 3). EMg was identified as the most important factor associated with bacterial communities (*R*^2^ = 0.0724, *p* < 0.01), whereas TN had the strongest effect on fungal communities (*R*^2^ = 0.0576, *p* < 0.01).

**Table 3:** The results of hierarchical partitioning (HP) based on soil bacterial (a) and fungal (b) community and soil factors.

Factor	Bactial		Fungal	
	Individual	*p*	Individual	*p*
TN	0.0723	0.01**	0.0576	0.005**
TP	0.048	0.01**	0.0445	0.005**
TK	0.0456	0.03*	0.0368	0.015*
SOM	0.0449	0.03*	0.0404	0.01**
EMg	0.0724	0.01**	0.0493	0.005**
ECa	0.0471	0.01**	0.029	0.02*
AFe	0.0534	0.02*	0.0431	0.005**
ACu	0.0445	0.03*	0.0397	0.005**
AMn	0.0356	0.04*	0.034	0.01**
AZn	0.0374	0.01**	0.0327	0.015*
AK	0.0467	0.02*	0.0383	0.01**
AP	0.044	0.01**	0.0481	0.02*
NN	0.04	0.02*	0.0288	0.015*
AN	0.0276	0.06	0.0323	0.01**
pH	0.0443	0.02*	0.0337	0.02*
EC	0.0431	0.01**	0.0293	0.005**

The significance thresholds corresponding to the asterisks in the table are as follows: *p < 0.05, **p < 0.01.

Hierarchical partitioning (HP) further showed that soil factors explained 58.7 and 28.7% of the variation in bacterial and fungal communities, respectively. Among these, the independent contributions of trace metal ions (0.113 for bacteria, 0.057 for fungi) were higher than those of soil nutrients (0.069 for bacteria, 0.022 fore fungi). Nevertheless, a substantial fraction of the variability was accounted for jointly by both sets of variables, reflecting interdependency among environmental factors.

Taken together, these results consistently suggest that trace metal ions play a more important role than soil nutrients in shaping microbial community structure, although their influence is somewhat intertwined with other environmental factors.

### 3.3 Co-occurrence networks of bacterial and fungal communities and their relationship with soil factors

**Figure 7:**
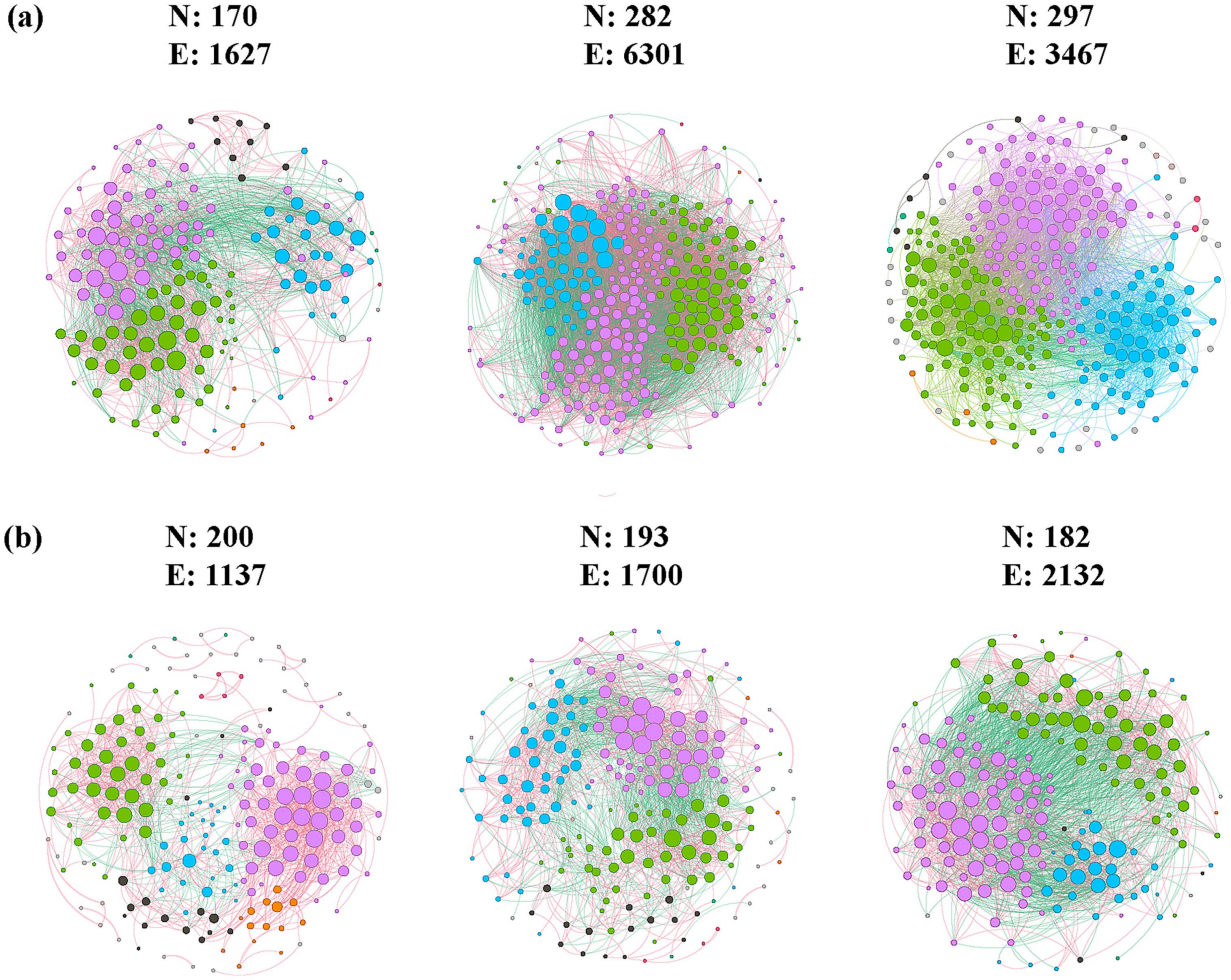
The co-occurrence networks for bacterial **(a)** and fungal **(b)** communities. N, node; E, edge. Node size is proportional to node degree. Positive links between nodes were colored green and negative links were colored red. Modularity class were randomly colored.

The complexity of bacterial co-occurrence networks exhibited a unimodal pattern along the altitude gradient, whereas fungal network complexity increased with altitude gradient (Supplementary Table S3; Figure 7). Several topological properties of bacterial networks, such as edge count, connectance, average degree, clustering coefficient, and degree centrality, initially increased and then decreased with increasing altitude gradient. In contrast, the fungal networks showed an increase in edge count, connectivity, and average degree with altitude gradient, while the average path length and diameter decreased, indicating that the fungal networks became more complex at higher altitude gradients.

Positive correlations were the dominant type of interaction between nodes in both bacterial (>52.28%) and fungal (>56%) networks, indicating that cooperative or facilitative interactions (e.g., cross-feeding, syntrophy) prevail in these soil microbial communities under ambient conditions (Li et al., 2020). In bacterial networks, the fraction of positive correlations initially decreased and then increased, showing an opposite trend to that of network complexity. This U-shaped pattern suggests that bacterial cooperative interactions are suppressed at mid-altitude gradient where environmental conditions (e.g., moderate disturbance or resource availability) may favor competitive over facilitative strategies. Conversely, the proportion of positive correlations in fungal networks decreased monotonically with altitude gradient, while the proportion of negative correlations correspondingly increased (Supplementary Table S3). This trend indicates that higher altitude gradients progressively reduce cooperative interactions among fungal taxa, while simultaneously enhancing competitive or niche-partitioning interactions. The altitude gradients-driven shift from cooperation to competition in fungal networks can be attributed to several mechanisms. First, increasing environmental stress at higher altitude gradients (e.g., lower temperature, shorter growing seasons, and reduced water availability) intensifies resource scarcity, forcing fungal taxa to compete for limited carbon substrates (Yang et al., 2021; He et al., 2024b). Second, the high environmental heterogeneity at high altitude gradient promotes niche differentiation among fungal taxa as a strategy to avoid direct competition, which manifests as increased negative associations in co-occurrence networks (Li et al., 2020). Third, the transition from mutualistic to more antagonistic plant-fungus relationships under cold and dry conditions may further contribute to the elevated proportion of negative interactions (Neat et al., 2026).

Multiple regression on matrices (MRM) analysis (Table 4) revealed that AK, AP, and TP were the most significant factors associated with bacterial network complexity, followed by AFe, NN, ACu, TK, EMg, SOM, AZn, and pH. For fungal networks, AZn, AK, TP, and AP were identified as important correlates, with ACu, pH, and NN also having significant effects. Stepwise regression analysis showed that soil nutrients explained a larger proportion of the complexity in bacterial networks (81.39%) compared to trace metal ions (48.36%), whereas for fungal networks, trace metal ions (86.31%) showed a higher contribution than nutrients (66.26%) (Supplementary Tables S4, S5).

**Table 4:** Multiple regression on matrices (MRM) analysis of soil bacterial and fungal network characteristics and soil factors.

Factor	Bactial		Fungal	
	R^2^	0.85***	R^2^	0.945***
TP	−0.107***		0.055***	
TN	−0.005		0.009	
TK	−0.048***		−0.011	
SOM	0.024**		−0.017	
pH	−0.018*		−0.026**	
NN	0.066***		0.016*	
AN	0.001		0.007	
EMg	−0.041***		−0.01	
ECa	0.016		−0.013	
EC	0.021		0.002	
AZn	−0.023*		0.232***	
AP	0.117***		−0.045***	
AMn	−0.006		0.009	
AK	0.187***		0.08***	
AFe	0.073***		0.007	
ACu	−0.055***		−0.038***	

*p < 0.05, **p < 0.01, ***p < 0.001.

### 3.4 Assembly processes of bacterial and fungal communities and their relationship with soil factors

**Figure 8:**
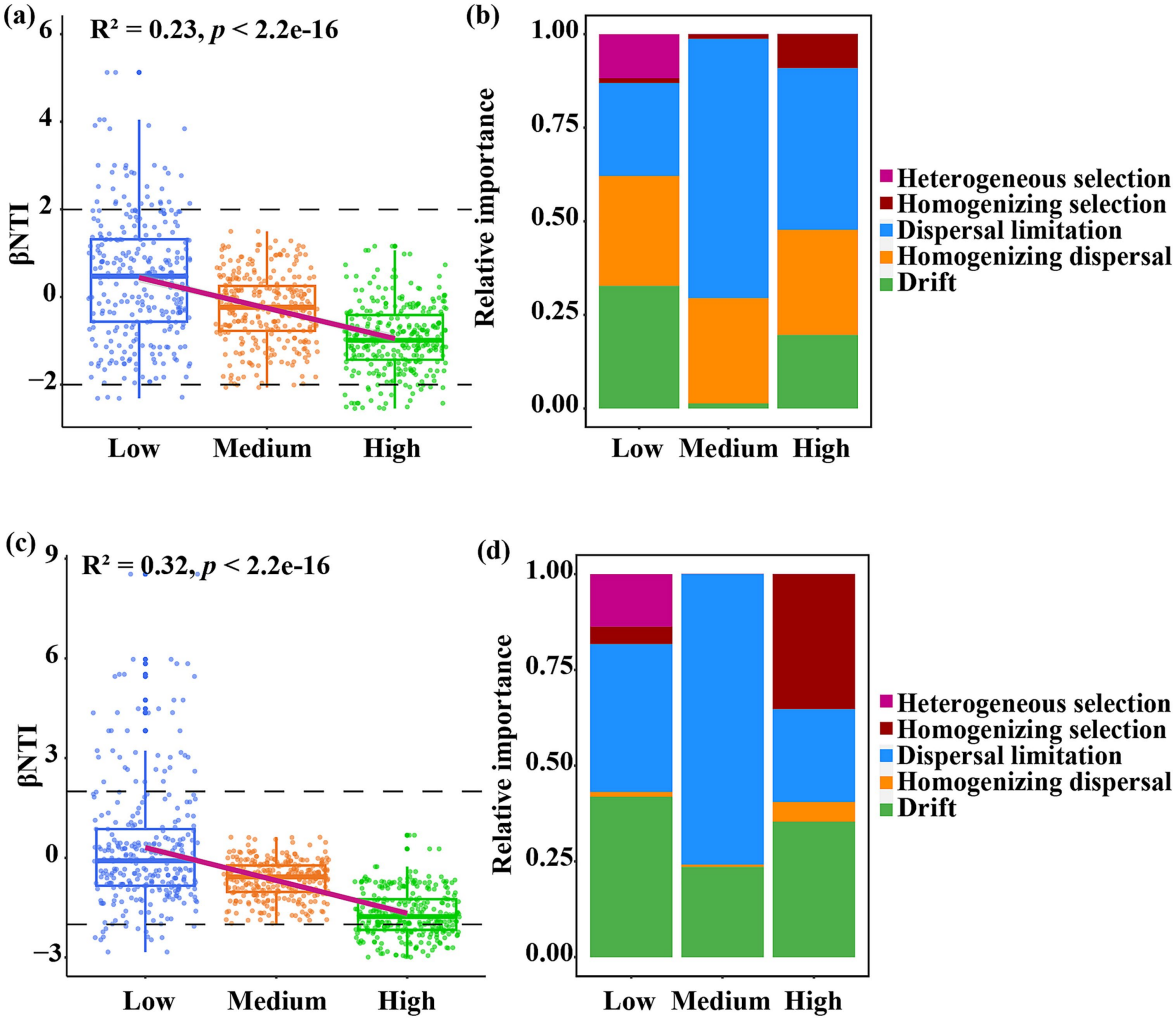
Assembly processes of bacterial and fungal communities. Linear relationships between altitude gradients and assembly processes (βNTI) of bacterial **(a)** and fungal **(c)**, the relative importance of assembly processes in bacterial **(b)** and fungal **(d)** community assembly across altitude gradients.

Using null-model-based statistical inference (βNTI and RCBray), the beta nearest taxon index (βNTI) values for both bacterial and fungal communities predominantly ranged between −2 and 2 across the altitude gradient (Figures 8a,c), indicating that stochastic processes were statistically inferred to play a dominated role in microbial community assembly in vineyard soils. Moreover, βNTI values were significantly negatively correlated with altitude gradient, suggesting that the inferred contribution of stochastic processes became more pronounced at higher altitude gradients.

The proportion of stochasticity in the assembly of bacterial and fungal communities exhibited a unimodal pattern, with the highest values observed at the medium altitude gradient (Figures 8b,d). Our null-model results further indicated that, as altitude gradient increased, bacterial communities shifted from being primarily influenced by drift to being governed by dispersal limitation and homogenizing dispersal, with dispersal limitation being the predominant process inferred. In contrast, fungal communities were driven mainly by drift at both low and high altitude gradients, while dispersal limitations were inferred to dominate at the medium altitude gradient. The effect of dispersal limitations on bacterial and fungal communities-as inferred from the null-model framework-initially increased and then decreased with altitude gradient. Statistically, this pattern reflects a shift in the inferred stochastic assembly mechanisms from birth-death processes (drift) toward dispersal limitation along the altitude gradient.

MRM analysis revealed that soil factors had a greater impact on bacterial assembly processes compared to fungal assembly processes (Table 5). The important correlates associated with bacterial assembly included ACu, AFe, AZn, EMg, AP, AMn, SOM, NN, and AN, with ACu, AP, and AN exerting significant negative effects, while the remaining factors exhibited positive influences. For the fungal community, ACu, AFe, pH, EMg, and NN were the most influential factors, with ACu and EMg negatively associated with fungal assembly. These results highlight the significant role of trace metal ions in shaping the assembly processes of both bacterial and fungal communities.

**Table 5:** Multiple regression on matrices analysis (MRM) of soil bacterial and fungal assembly processes and soil factors.

Factors	Bacterial		Fungal	
	R^2^	0.557***	R^2^	0.195**
	Int	0.553***	Int	−0.689**
TP	0.132		−0.121	
TN	0.132		−0.388	
AP	−0.428***		0.112	
AK	−0.12		−0.15	
pH	0.212		0.375*	
AZn	0.675***		0.025	
ECa	0.169		0.056	
EMg	0.543***		−0.359*	
EC	0.038		0.117	
AFe	0.836***		0.822***	
ACu	−1.163***		−0.825***	
AMn	0.39***		0.015	
TK	−0.058		0.045	
SOM	−0.367**		−0.185	
NN	0.355***		0.332*	
AN	−0.182**		−0.145	

*p < 0.05, **p < 0.01, ***p < 0.001.

## 4 Discussion

### 4.1 Bacterial and fungal diversity and community composition along altitude gradients and their important correlates

It is important to note that the altitude gradient is not a direct mechanistic driver, but rather a composite proxy that incorporates multiple co-varying environmental factors, including temperature, moisture, radiation, soil properties, and potentially management intensity. Therefore, in this section we interpret the observed patterns as being associated with a suite of environmental changes occurring along the altitude gradient, rather than attributing them to the altitude gradient per se.

The bacterial community composition was significantly correlated with the altitude gradient. Consistent with typical soil communities, *Actinobacteria*, *Proteobacteria*, and *Acidobacteria* were the dominant bacterial phyla within the soil ecosystem (Morrissey et al., 2019). In this study, fungal communities were dominated by *Ascomycota*, which aligns with previous findings from the Tibetan Plateau (Zhang et al., 2013; Hussain et al., 2021). *Ascomycota* have a superior ability to decompose cellulose compared to other fungal groups (Štursová et al., 2012). Importantly, the higher abundance of *Ascomycota* at higher altitude gradients coincided with significantly higher soil organic matter (SOM) there (Supplementary Table S6), suggesting that SOM, rather than altitude gradient itself, is a key correlate of this fungal phylum’s distribution.

Globally, no consistent pattern exists for how soil microbial diversity is geographically distributed along altitude gradients (Wang et al., 2019). Although some research suggests a monotonically decreasing trend with altitude gradient (Looby and Martin, 2020), the diversity indices of bacteria and fungi in this study mostly presented a unimodal pattern. This variation is likely associated with the interplay of climate conditions, soil nutrients, and trace metal ions that co-vary with altitude gradients (Supplementary Table S6). While environmental conditions can become harsher at higher altitude gradients in many mountains (Margesin et al., 2009), potentially leading to diversity declines, we found that soil nutrients (TN, SOM, TP) significantly increased along the altitude gradients in our study system (Supplementary Table S6), which is known to promote microbial growth (Peay et al., 2017). This positive nutrient trend, together with other covariates, helps explain the observed unimodal diversity pattern. Such unimodal patterns have also been reported in previous studies (Ren et al., 2018; Zhao et al., 2020; Vélez-Martínez et al., 2024). In addition, we observed that different diversity indices can exhibit contrasting patterns: for example, the bacterial Chao 1 index exhibited an unimodal pattern with increasing altitude gradient, while the bacterial Shannon index showed a significant monotonic decreasing pattern. Therefore, it is essential to specify which diversity index to use when describing the geographical distribution of microbial diversity along an altitude gradient.

The distance-decay patterns of bacterial and fungal communities revealed that bacteria exhibited a higher spatial turnover rate than fungi. Moreover, network analysis and assembly processes indicated that bacterial communities changed more markedly and were more sensitive to climatic (or spatial) factors that co-vary with altitude gradients than fungal communities. HP and MRM analyses further demonstrated that soil environmental factors have a higher explanatory power for variations in fungal community composition, network complexity, and assembly processes. Collectively, these findings highlight that bacterial communities are more sensitive to environmental changes associated with altitude gradients than fungal communities.

Previous studies have identified pH as a key factor correlated with changes in bacterial community composition across altitude gradients (Ji et al., 2022; Liu et al., 2021). In this study, however, pH had a relatively minor influence on the bacterial community composition in the vineyard soils across the altitude gradient. This may be related to human interventions such as soil improvement, fertilization and weeding, which may buffer the natural pH gradient. Instead, Mg^2+^ emerged as a strongly associated factor with bacterial community composition, potentially playing a crucial role in bacterial growth and survival (Dong-yeon et al., 2019). Research has shown that varying magnesium concentrations can alter the overall structure of agricultural soils (Hou et al., 2021). Total nitrogen (TN) was the most significant factor associated with fungal community composition, consistent with previous studies (Han et al., 2024). As a key element for plants and microorganisms, the significant increase in TN with altitude gradients in this study is likely to provide the fungus with the necessary resources to withstand the harsher environmental conditions found at higher altitude gradients.

### 4.2 Inconsistent variations in bacterial and fungal network characteristics with altitude gradient

Microbial communities are complex assemblages of highly interacting taxa. In this study, bacterial network complexity followed an unimodal pattern along the altitude gradient, with higher edge numbers and average degrees at the medium altitude gradient. This pattern is most likely related to specific environmental conditions characteristic of the mid-altitude gradient. For example, the mid-altitude gradient regions typically experience moderate environmental disturbances or stresses (such as fluctuations in moisture and temperature), which neither completely destroy the microbial community structure nor allow dominance by a few species, potentially promoting interactions among multiple species (Duan et al., 2025). Additionally, the mid-altitude gradient zone is often a region where deterministic processes (environmental selection) and stochastic processes (diffusion limitation, ecological drift) coexist in equilibrium, a state that may be conducive to forming complex interaction networks (Zhang et al., 2025). Similar findings have been reported in agricultural soils of *Camellia oleifera* plantations (Zhang et al., 2025).

In contrast, the complexity of the fungal network increased steadily with altitude gradient. This pattern is consistent with the view that fungi are more resilient than bacteria to environmental stresses (Liu Y et al., 2024), potentially strengthening their interactions to counteract these stresses. de Vries et al. (2018) also found that fungal networks are more stable than bacterial ones under extreme conditions, a finding that may be due to fungi’s greater resistance and tolerance to low-temperature soil environments as reported in other systems (Kirchman, 2018). While biologically plausible, we did not directly measure fungal cold tolerance in this study; thus, this interpretation should be considered as a hypothesis rather than a demonstrated conclusion.

Both bacterial and fungal networks were primarily characterized by positive associations, highlighting microbial synergies. However, the fraction of negative correlations within the fungal network increased with altitude gradient. One possible explanation is that competitive exclusion or ecological niche differentiation becomes more important at higher altitude gradients, although correlation-based network analysis alone does not directly demonstrate such ecological interactions. Among the soil properties measured, available potassium (AK), available phosphorus (AP), and total phosphorus (TP) were the primary factors associated with the complexity of both bacterial and fungal networks. In this study, the AK, AP, and TP contents increased significantly with altitude gradient, thus showing a strong correlation with microbial network complexity.

### 4.3 Assembly processes of bacterial and fungal communities

Growing evidence suggests that spatial variables can explain changes in community composition (e.g., via dispersal limitation), and that neutral processes can govern the assembly of bacterial and fungal communities (Lindström and Langenheder, 2012; Sloan et al., 2006). Using null-model-based statistical inference (βNTI and RCBray), our findings reveal that stochastic processes dominated the assembly of both bacterial and fungal communities along the altitude gradients, consistent with previous reports (Hussain et al., 2021; Ji et al., 2024). One possible interpretation is that the extreme and heterogeneous environmental conditions often found in alpine ecosystems may lead environmental pressures to outweigh the effects of interspecies competition and selection, potentially causing communities to rely more on dispersal limitation and drift (Zhou and Ning, 2017). However, it is important to acknowledge that these inferred ecological processes-drift, dispersal limitation, homophily selection, etc.-are statistically derived from null-model framework rather than directly observed. Therefore, the following explanation should be considered as an assumption informed by our data, rather than as a directly demonstrated mechanism.

It is generally believed that environmental heterogeneity increases with geographic distance (Zhang et al., 2022), which also explains the distance-decay patterns we observed. The high proportion of dispersal limitation and drift during community assembly could be explained by: first, soil bacteria and fungi in the high-altitude gradient areas may tend toward dormancy to cope with harsh conditions (Nemergut et al., 2013); second, prolonged soil freezing can enhance physical barriers, increasing dispersal limitations; third, freeze–thaw cycles may cause disturbances, leading to ecological drift (Kang et al., 2024).

At lower altitude gradients, bacterial communities were primarily influenced by drift. As altitude gradient increased, it is plausible that harsher environmental conditions often associated with higher altitude gradients-such as lower temperatures and reduced oxygen, although these variables were not directly measured in this study-may contribute to limited microbial dispersal (Kang et al., 2024), with dispersal limitation emerging as the dominant process in our statistical inference. We therefore present this interpretation as a hypothesis that requires direct validation in future studies. Fungal communities at both low and high altitude gradients were primarily governed by drift, while dispersal limitation dominated at the medium altitude gradient, a pattern that may be tentatively attributed to fungi’s superior ability to adapt to extreme environments, as suggested by previous studies (Liu Y et al., 2024). However, because we did not directly measure fungal physiological traits or stress tolerance, this interpretation remains speculative and should be tested further. The MRM results indicated that ACu and AFe were the most significant factors associated with bacterial and fungal assembly processes. These findings are consistent with the view that trace metal ions are critical correlates of microbial-driven processes (Dai et al., 2023), potentially influencing not only diversity and network characteristics but also the fundamental processes by which communities assemble.

### 4.4 Limitations and caveats

We acknowledge several important limitations. First, as noted throughout the discussion, the altitude gradient is a composite proxy for multiple covariate environmental factors. Our study design, with only three vineyards sampled per altitude gradient class, does not allow us to statistically disentangle the individual effects of altitude gradients themselves from other correlated variables such as microclimate and management practices. Therefore, the associations we report should be interpreted as correlations with the entire suite of environmental changes that occur along the altitude gradient, rather than as causal effects of the altitude gradient. Second, the co-occurrence network analysis presented here was based on abundance correlations, which do not directly demonstrate ecological interactions such as competition, facilitation, or adaptation. Therefore, our interpretation of negative correlations as potential indicators of competitive exclusion or niche differentiation should be considered as hypotheses or possible explanations, rather than as demonstrated ecological mechanisms. Third, the potential for confounding due to unmeasured vineyard-specific characteristics (e.g., varietal differences, agronomic practices) remains. Future studies with a larger number of independent sites per altitude gradient level and replicated altitude gradients in different regions are needed to confirm the robustness of our findings and to better separate the effects of natural environmental filters from those of human management.

Moreover, it is important to emphasize that our analysis is relevant. Although trace metal ions show a strong association with microbial community variability, causality cannot be inferred directly from HP or MRM results. Controlled experiments or long-term observations are needed to validate the mechanistic role of trace metal ions. Readers should interpret effect sizes (e.g., *R*^2^) as measures of explanatory power rather than evidence of causation.

Additionally, while we have attempted to account for vineyard-level clustering by using plot-level analyses (*n* = 54) and conducting conservative checks with vineyard-mean values (*n* = 9), our study design includes only three vineyards per altitude gardient class. Future studies with a larger number of independent vineyards per altitude gradient level would be valuable to more rigorously disentangle altitude gradient effects from vineyard-specific features.

In interpreting our results, we note that statistical significance and effect size should be weighed together. For example, the *R*^2^ values reported in dbRDA and MRM analyses reflect the proportion of variance explained; even modest *R*^2^ values may be ecologically meaningful given the complexity of soil systems.

## 5 Conclusion

This study investigated the spatial vertical distribution patterns of soil microbial communities in vineyards under anthropogenic management and identified the important correlates influencing microbial community structure. Our findings reveal that, with the exception of the increase in fungal network complexity, which increases with altitude gradient, microbial diversity, network complexity, and assembly processes each exhibit unimodal patterns along altitude gradient. Changes in soil properties emerged as the primary factors associated with bacterial (EMg) and fungal (TN) community composition and diversity. Network analysis further suggests that fungi may possess greater environmental adaptability than bacteria, which may be linked to an increased proportion of negative correlations among fungal taxa, potentially reflecting intensified competitive interactions or niche differentiation that may enhance fungal resistance to harsh environmental conditions. The stochastic nature of microbial community assembly processes in vineyard soils was motivated by the extreme and heterogeneous conditions typical of alpine ecosystems. It is important to note that the bacterial communities exhibited greater sensitivity to altitude gradients compared to the fungal communities. Additionally, trace metal ions showed stronger explanatory power in shaping microbial composition, diversity, networks, and assembly processes than soil nutrients in this study. Together, these findings advance our understanding of how soil microbial communities respond to altitude gradients in managed vineyard systems and may inform future strategies for sustainable soil management in similarly fragmented agricultural landscapes.

## Data Availability

The original contributions presented in the study are included in the article/Supplementary material, further inquiries can be directed to the corresponding authors.

## Footnotes

1
http://gephi.github.io/
