# Supplementary material for: Trace metal ions are strongly associated with the structural variation in vineyard soil microbial communities along altitude gradients
Source: Front Microbiol. 2026 Jul 8;17:1824682. doi: 10.3389/fmicb.2026.1824682 (PMC13388906; doi:10.3389/fmicb.2026.1824682)
Supplement: Supplementary file 1 [file Table_1.DOCX]

**Supporting materials**

Table S1 Significance analysis of the relative abundance of soil bacterial and fungal phyla across different altitude gradients

| Phylum | | Hight | Medium | Low |
| --- | --- | --- | --- | --- |
| Bacterial | Actinobacteria | 30.11±2.61a | 33.59±6.27a | 31.57±9.45a |
|  | Proteobacteria | 28.46±1.31a | 27.02±2.73a | 29.19±5.13a |
|  | Acidobacteria | 15.95±2.07a | 15.88±3.40a | 14.42±2.91a |
|  | Chloroflexi | 11.97±1.52a | 10.33±1.32b | 11.93±1.16a |
|  | Gemmatimonadetes | 1.84±0.24c | 2.88±0.43b | 3.74±0.65a |
|  | Rokubacteria | 2.16±0.61ab | 2.51±0.73a | 1.71±0.78b |
|  | Firmicutes | 2.69±0.53a | 1.23±0.28c | 2.22±0.67b |
|  | Bacteroidetes | 2.41±0.40a | 1.51±0.39b | 1.33±0.67b |
|  | Entotheonellaeota | 1.26±0.16a | 0.82±0.22b | 0.56±0.15c |
|  | Planctomycetes | 0.71±0.39b | 1.19±0.64a | 0.71±0.48b |
|  | Others | 2.46±0.41b | 3.05±0.54a | 2.63±0.70b |
| fungal | Ascomycota | 87.78±1.78a | 80.02±5.46b | 79.81±9.04b |
|  | Mortierellomycota | 5.46±1.67a | 5.42±2.57a | 5.03±3.12a |
|  | Basidiomycota | 2.32±0.68b | 5.13±2.27a | 4.24±3.15a |
|  | Glomeromycota | 0.01±0.01a | 0.35±0.44a | 0.16±0.25a |
|  | Olpidiomycota | 0.20±0.20a | 0.19±0.20a | 0.04±0.05a |
|  | Zoopagomycota | 0.06±0.05ab | 0.07±0.05a | 0.05±0.05b |
|  | Mucoromycota | 0.01±0.01a | 0.01±0.01a | 0.07±0.07a |
|  | Chytridiomycota | 0.04±0.02a | 0.03±0.04a | 0.02±0.02a |
|  | Rozellomycota | 0.02±0.02a | 0.01±0.02a | 0.04±0.12a |
|  | Blastocladiomycota | 0.00±0.01a | 0.00±0.00a | 0.05±0.17a |
|  | Others | 4.11±0.97b | 8.86±3.84a | 10.52±3.98a |

Table S2 Significance analysis of the relative abundance of soil bacterial and fungal genus across different altitude gradients

| Genus | | Hight | Medium | Low |
| --- | --- | --- | --- | --- |
| Bacterial | Subgroup_6 | 10.88±1.40a | 9.94±2.44a | 7.14±1.78b |
|  | 67-14 | 2.42±0.28b | 3.83±1.19a | 3.68±1.25a |
|  | KD4-96 | 3.12±0.76a | 3.24±0.58a | 2.52±0.40b |
|  | Rokubacteriales | 2.16±0.61ab | 2.51±0.73a | 1.71±0.78b |
|  | Solirubrobacter | 1.22±0.18b | 2.28±0.54a | 2.65±0.94a |
|  | Gaiella | 2.71±0.28a | 2.17±0.28b | 1.23±0.43c |
|  | MND1 | 1.98±0.39b | 2.38±0.39a | 1.47±0.63c |
|  | Pseudonocardia | 1.32±0.32b | 1.30±0.16b | 1.83±0.45a |
|  | Skermanella | 2.11±0.54a | 1.35±0.74b | 0.76±0.36c |
|  | RB41 | 0.43±0.20b | 0.93±0.18b | 2.82±1.66a |
|  | Others | 71.66±2.80b | 70.08±2.73b | 74.18±2.18a |
| fungal | Fusarium | 4.65±2.44b | 9.34±2.94a | 12.21±6.98a |
|  | Gibberella | 15.54±4.29a | 8.43±5.68b | 2.23±1.58c |
|  | Mortierella | 5.45±1.67a | 5.42±2.57a | 5.00±3.13a |
|  | Botryotrichum | 4.40±2.32a | 3.79±2.60a | 2.73±3.15a |
|  | Preussia | 3.06±1.84a | 2.99±3.38a | 3.35±4.24a |
|  | Lophotrichus | 4.89±4.48a | 0.78±0.54b | 3.05±5.52ab |
|  | Humicola | 2.33±1.37b | 3.48±1.28a | 1.84±1.65b |
|  | Staphylotrichum | 1.72±1.04ab | 2.18±1.03a | 1.09±1.05b |
|  | Ilyonectria | 1.87±0.63b | 2.35±0.70a | 0.62±0.75c |
|  | Acaulium | 1.34±1.69a | 0.40±0.53a | 2.94±5.43a |
|  | Others | 54.75±8.57b | 60.88±5.43a | 65.10±8.38a |

Table S3 The topological properties for soil bacterial and fungal co-occurrence networks in different attitude gradients

|  | Bacteria | | | Fungi | | |
| --- | --- | --- | --- | --- | --- | --- |
|  | Low | Medium | High | Low | Medium | High |
| Nodes | 170 | 282 | 297 | 200 | 193 | 182 |
| Edges | 1627 | 6301 | 3467 | 1137 | 1700 | 2132 |
| Positive edges | 982  (60.36%) | 3294  (52.28%) | 1879  (54.2%) | 989  (86.98%) | 956  (56.24%) | 1194  (56%) |
| Negative edges | 645  (39.64%) | 3007  (47.72%) | 1588  (45.8%) | 148  (13.02%) | 744  (43.76%) | 938  (44%) |
| Connectance | 0.113 | 0.159 | 0.079 | 0.057 | 0.092 | 0.129 |
| Average degree | 19.141 | 44.688 | 23.347 | 11.37 | 17.617 | 23.429 |
| Average path length | 2.052 | 1.679 | 2.254 | 2.746 | 2.118 | 1.849 |
| Diameter | 5.906 | 4.437 | 6.365 | 7.846 | 6.788 | 4.504 |
| Average clustering coefficient (Average.CC) | 0.601 | 0.627 | 0.56 | 0.629 | 0.569 | 0.607 |
| No clusters | 2 | 2 | 5 | 14 | 6 | 2 |
| Degree Centrality | 0.189 | 0.268 | 0.208 | 0.149 | 0.205 | 0.185 |
| Betweenness Centrality | 0.043 | 0.062 | 0.089 | 0.173 | 0.061 | 0.069 |
| Centralization closeness | 1.251 | 1.117 | 1.275 | 1.2 | 1.203 | 1.179 |
| Relative modularity (RM) | 2.291 | 3.798 | 2.267 | 1.394 | 1.199 | 2.233 |
| Modularity | 0.483 | 0.448 | 0.498 | 0.537 | 0.353 | 0.456 |
| Modularity random | 0.147 | 0.093 | 0.152 | 0.224 | 0.161 | 0.141 |

Table S4 Multiple regression on matrices analysis of soil bacterial and fungal network characteristics and soil nutrients

| Factors | Bacterial | | Fungal | |
| --- | --- | --- | --- | --- |
|  | R^2^ | 0.814*** | R^2^ | 0.663*** |
| TP | -0.073*** | | -0.023 | |
| TN | 0.009 | | 0.093*** | |
| TK | -0.051*** | | -0.054* | |
| SOM | 0.025** | | -0.035 | |
| AP | 0.095*** | | 0.016 | |
| NO_3_^-^-N | 0.034*** | | 0.005 | |
| NH_4_^+^-N | -0.008 | | 0.093*** | |
| AK | 0.157*** | | 0.166*** | |

Table S5 Multiple regression on matrices analysis of soil bacterial and fungal network characteristics and soil trace metals

| Factors | Bacterial | | Fungal | |
| --- | --- | --- | --- | --- |
|  | R^2^ | 0.484*** | R^2^ | 0.863*** |
| EMg | 0.022* | | 0.035** | |
| ECa | 0.078*** | | -0.015 | |
| AZn | 0.102*** | | 0.248*** | |
| AMn | 0.019* | | -0.001 | |
| AFe | -0.06*** | | 0.002 | |
| ACu | 0.025* | | -0.042** | |

Table S6 Physicochemical properties of soils with different altitude gradients

| Physicochemical indicators | High altitude | Medium altitude | Low altitude |
| --- | --- | --- | --- |
| pH | 7.79±0.09c | 8.00±0.09b | 8.22±0.11a |
| EC (μS·cm^-1^) | 212.07±19.30a | 137.47±25.02b | 102.89±6.61c |
| SOM (g·kg^-1^) | 61.71±13.31a | 76.11±34.04a | 9.15±0.06b |
| TN (g·kg^-1^) | 4.31±0.28a | 3.78±0.15b | 0.42±0.03c |
| TP (g·kg^-1^) | 1.07±0.24a | 0.72±0.17b | 0.27±0.03c |
| TK (g·kg^-1^) | 14.08±1.59a | 14.23±2.04a | 4.96±0.39b |
| AK (mg·kg^-1^) | 131.15±29.7a | 57.83±2.83b | 34.77±0.47c |
| AP (mg·kg^-1^) | 37.66±5.04a | 13.38±6.15b | 6.00±0.41c |
| NO_3_^-^-N (mg·kg^-1^) | 9.02±0.96b | 11.75±3.89a | 2.87±0.22c |
| NH_4_^+^-N (mg·kg^-1^) | 5.20±3.89a | 6.66±2.78a | 1.93±0.17b |
| EMg (mg·kg^-1^) | 244.65±17.32a | 193.28±16.13b | 104.59±2.11c |
| ECa (g·kg^-1^) | 4.53±0.17a | 4.07±0.22b | 3.8±0.05c |
| AFe (mg·kg^-1^) | 9.09±2.67a | 10.84±0.93b | 2.19±0.22c |
| ACu (mg·kg^-1^) | 3.97±2.11a | 4.87±1.94b | 0.47±0.04c |
| AMn (mg·kg^-1^) | 7.57±2.06b | 27.88±16.8a | 2.33±0.37b |
| AZn (mg·kg^-1^) | 8.23±4.6a | 4.13±0.99b | 0.81±0.1c |
